# A Customer Behavior Recognition Method for Flexibly Adapting to Target Changes in Retail Stores

**DOI:** 10.3390/s22186740

**Published:** 2022-09-06

**Authors:** Jiahao Wen, Toru Abe, Takuo Suganuma

**Affiliations:** 1 Graduate School of Information Sciences, Tohoku University, 6-3-09 Aramaki-Aza-Aoba, Aoba-ku, Sendai 980-8579, Japan; 2 Cyberscience Center, Tohoku University, 6-3 Aramaki-Aza-Aoba, Aoba-ku, Sendai 980-8578, Japan

**Keywords:** smart retail, in-store camera, customer behavior recognition, behavior reconstruction

## Abstract

To provide analytic materials for business management for smart retail solutions, it is essential to recognize various customer behaviors (CB) from video footage acquired by in-store cameras. Along with frequent changes in needs and environments, such as promotion plans, product categories, in-store layouts, etc., the targets of customer behavior recognition (CBR) also change frequently. Therefore, one of the requirements of the CBR method is the flexibility to adapt to changes in recognition targets. However, existing approaches, mostly based on machine learning, usually take a great deal of time to re-collect training data and train new models when faced with changing target CBs, reflecting their lack of flexibility. In this paper, we propose a CBR method to achieve flexibility by considering CB in combination with primitives. A primitive is a unit that describes an object’s motion or multiple objects’ relationships. The combination of different primitives can characterize a particular CB. Since primitives can be reused to define a wide range of different CBs, our proposed method is capable of flexibly adapting to target CB changes in retail stores. In experiments undertaken, we utilized both our collected laboratory dataset and the public MERL dataset. We changed the combination of primitives to cope with the changes in target CBs between different datasets. As a result, our proposed method achieved good flexibility with acceptable recognition accuracy.

## 1. Introduction

Smart retail is regarded as an arrangement of the Internet of Things and big data analytics for retail purposes [1]. Usually, it collects data from videos captured by ubiquitous cameras in retail stores. Consequently, we need to extract valuable information collected by videos. Customer behavior (CB) is commonly considered to be a kind of valuable analytic material for business management [2]. As there are an almost infinite number of classes of CBs in retail environments, generally, specific CBs are selected as recognition targets, called target CBs, based on needs. Typically, customer-centric retailing demands different target CBs to analyze the customer decision-making process. Usually, the target CB changes frequently with different products or in-store layouts because of the different customer-product interactions. For instance, trying on clothes in a clothes shop, sitting on a bed in a furniture shop, picking up a bottle from the shelf, picking up an ice cream from a freezer, etc. Accordingly, CB recognition (CBR) methods should be modified to recognize the changed target CBs. In some cases, a current target CB is required to be discriminated, e.g., in the case of “pick a product”, discriminating whether a customer is picking a product with one hand or both hands provides information regarding the customer’s effort to pick a product. Therefore, a CBR method is expected to be flexible enough to address the issue of frequent changes in the target CB.

As CBR is a branch of human activity recognition (HAR), current CBR methods use machine learning (ML)-based models [3] due to their remarkable accuracy in HAR tasks. Nevertheless, in contrast to human activity recognition, CBR methods also require flexibility. For frequent target CB changes, to recognize different target CBs, namely, changing the model’s output, ML-based models require time-consuming re-collection of training data and training the model. Though transfer learning can be applied in some cases for faster training, the inevitable step of data collection is still time-consuming. This causes current methods to be inflexible when coping with changes in target CBs. Additionally, in existing methods, target CBs are mostly selected arbitrarily according to the training data, instead of business needs, which indicates that change adaptation is not considered in their design. Thus, current CBR methods are not suitable for target CBR tasks in retail environments.

To cope with target changes, we propose a rule-based method to recognize CB by the combination of primitives, each of which is a kind of partitioned unit of CB. Since primitives are allowed to be combined for the customization of various CBs, our proposed method can reuse the primitives to customize the changed target CBs. The number of combinations of primitives increases exponentially as the number of primitives increases linearly. Thus, our method can cover a wide range of CBs with a small number of primitives. As CB analysis focuses on customer-product interaction, we designed the primitive as a unit that describes an object’s motion or the relationship between multiple objects.

To conclude, rather than accuracy improvement, we focus on the method’s flexibility, which is also important in CBR requirements. Consequently, the main contribution of the paper is the proposal of a flexible CBR method to cope with frequent changes in target CBs.

We evaluated our method on our self-collected laboratory dataset and the public MERL dataset. Compared to the time-consuming collection of data and training of models, our method was able to deal with target changes in a short time, which implies its enhanced flexibility. Moreover, assessment of acceptable recognition accuracy indicated that we did not lose too much accuracy as the cost of achieving a high degree of flexibility.

The remainder of this paper is organized as follows: Section 2 explains the problems of existing methods in terms of their methodology and rationale for selecting target CBs. Section 3 describes our proposal of CB decomposition and the matching of CB patterns in detail. In Section 4, the evaluation of the performance of the proposed method on two different datasets is described. Finally, Section 5 concludes the paper with some final remarks and suggestions for future research.

## 2. Related Work

In retail environments, we analyze CBs to meet the demands of customer-centric retailing. As a result, CBR tasks should not only address the issues of methodology but also consider the difficulty of application and the customer’s experience. Currently, various types of sensors are used in HAR research to acquire data on human movements. In contrast, almost all research on CBR uses visual data. The major reason is that visual data-based approaches can be directly applied to video acquired by surveillance cameras in the store, which makes the application of these approaches hardware-free and avoids active customer participation [2]. In addition, visual data contains much more information than most other types of sensor data.

With the input of videos, existing CBR methods mainly use the pipeline of extracting features from consecutive frames within a certain period and recognizing behavior from the sequenced features using machine-learning-based models, especially the hidden Markov model (HMM). Popa et al. [4] proposed an HMM-based model to recognize customer’s buying behavior with optical flow features. Within the next two years, they improved the HMM-based model by partitioning the CB into basic actions [5], which are similar to our proposed primitives. However, they determined the basic actions by optical flow features. Thus, the model is not explainable, which results in it having poor flexibility when dealing with target CB changes. Djamal Merad et al. [6] applied an HMM model for hand movement analysis and an SVM model as eye-tracking descriptors for the classification of a customer’s purchasing type. The specific CB classes were not given because the authors conducted CBR indirectly. Moreover, their wearable device was difficult to apply to every customer, and required customers’ active participation. However, people are generally reluctant to cooperate without tangible rewards [2].

Apart from HMM models, convolutional neural networks (CNNs) are also widely used due to their excellent performance on spatial feature extraction. Singh et al. [7] used a CNN connected with a long short-term memory (LSTM) [8] model to recognize CBs, such as hand in the shelf, inspecting the products, etc. Using this method, Singh et al. avoided most object occlusions using top-view cameras. Some improved CNN-based models [3,9] have recently been proposed to detect customers and recognize basic customer-product interactions, such as picking up products, returning products back to the shelf, etc. Jingwen Liu et al. [10] employed a dynamic Bayesian network to conduct CBR of six CBs, including turning to shelf, touching, picking, returning, etc., based on hand movements and the orientation of the head and body. Jumpei Yamamoto et al. [11] estimated CB class in a book store based on depth features from a top-view camera and pixel state analysis (PSA) features using a support vector machine (SVM).

In addition, several studies, not using an ML-based model [12,13], implemented a complete CBR system with an RGB-D camera. Basic CBs, such as pick, return, etc., were recognized, based mainly on processing depth information by background subtraction. Unfortunately, since the systems were designed for specific purposes using simple and efficient methods, their flexibility was compromised.

In sum, although the aforementioned ML-based methods achieved improvements in CBR accuracy, they share common limitations with respect to flexibility, as follows:

- Difficulty in adapting to changes in target CBs: The ML models cannot be reused as long as the changed CBs are substantially different from the training data. In this event, time-consuming new training data collection and model re-training are required, which implies inflexibility.
- The model is not explainable: Unexplainable models can only be tuned based on their outputs. This implies poor flexibility during any modifications caused by changes in business needs.

Furthermore, since there are few approaches similar to our method in the field of CBR, we discuss the similarities and differences of several HAR methods with our approach with respect to their application to CBR. Liu et al. [14] proposed an HMM-based method which divides human activity into several phases, called “motion units”, analogous to phonemes in speech recognition. Yale et al. [15] proposed interpretable high-level features based on motion units. Different activities sharing the same motion units allow the model to derive more explanatory power from human activities. Although motion units are similar to our proposed primitives, the methods encounter two issues when applied to CBR tasks, which highlight how they differ. Firstly, these methods use data from a smartphone’s acceleration sensor. Alhough providing tangible rewards is less of a problem, the methods require the active participation of customers, e.g., downloading an app and agreeing to its terms of service, which increases saliency to customers. Consequently, the rewards increase the cost and the active participation creates privacy issues [2]. Secondly, despite the fairly complete categorization of human activities based on motion units, the methods do not focus on human-item interactions. Since purchase behavior can be easily detected from cashier records, recognizing non-purchase CB becomes one of the objectives of CBR. As the main component of non-purchase CBs, human-item interactions are required in CBR tasks. As an illustration, “picking up a product” and “returning a product” would be practically identical due to their similar hand motions. Nishant Rai et al. [16] divided human activities in indoor living spaces into atomic actions, analogous to the primitives in this paper. The use of both visual and audio data avoided users’ active participation, and the training data included human-item interactions. The authors improved recognition accuracy by training the model with annotations of both atomic actions and human activities. In contrast, we concentrated on improving the method’s flexibility without sacrificing too much accuracy, as flexibility is one of the important factors for CBR tasks. Romany F.Mansour et al. [17] combined a faster RCNN and a deep Q network for the detection of anomalous entities or human activities in videos. Since this is a typical ML-based HAR method, it requires re-collecting training data and re-training models to adapt to the changed recognition targets, which is inflexible for CBR tasks. In conclusion, the HAR methods described require major modifications before they could be applied to CBR tasks.

## 3. Proposal

**Figure 1:**
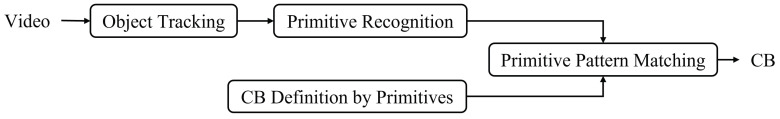
Proposal flow.

In this paper, we designed a unit, called a primitive, which is a kind of partitioned CB. Our CBR process consists of object tracking, primitive recognition, and CBR by matching recognized primitives with a predefined pattern of primitives. Since the innovative part of our approach is CBR with the combination of primitives, we applied existing methods to object tracking. The workflow of our approach is shown in Figure 1. At the beginning, the existing method tracks objects from the input video captured by in-store cameras. Then, each frame’s primitives are recognized based on the object trajectories. We predefine CB as a pattern consisting of primitives. Finally, we match the recognized primitives with the predefined primitive pattern. The matched pattern is regarded as the corresponding CB. This section explains our proposed method in detail, including how we design the primitives, the method for primitive recognition, customizing CB using primitives, and CBR by pattern matching.

### 3.1. Primitive

The dictionary definition of a behavior is the accomplishment of a thing, usually over a period of time or in stages. We believe that this definition reveals the process by which the human brain recognizes a behavior from visual information. Behavior consists of several stages, and our brains recognize this behavior by checking whether these stages occur in the correct order. In this paper, we refer to these stages as primitives. Thus, CB can be decomposed into primitive(s). Table 1 lists the target CBs in existing methods and the primitives from our subjective decomposition of the target CBs. We did not list a type of CB [18] in Table 1 because they recognize customer’s emotion from facial expressions and speech text, which might breach customers’ privacy. During the decomposition, we controlled the decomposition granularity to avoid redundancy from over-decomposition. We found that the objects in the target CBs were body parts or products. There are two types of primitives: one describes an object’s motion state and the other describes the relationship between two objects. Based on what we have found so far, we can decide what kind of information is in the primitive and how detailed it is.

It is necessary to design an expression format for primitives. Generally, using natural language is considered an efficient method when we need to let others know that we understand a behavior. Therefore, we define the primitive by a sentence with reference to the natural language grammar. The syntax is:

(1) $$subject\;verb\;object\;from\;where_{start}\;to\;where_{end},$$

where italic words are syntax elements which can be replaced by words in the vocabulary below. If $where_{start}=where_{end}$, the syntax can be simplified as subjectverbobjectwhere. As the syntax shows, the primitive consists of subject, verb, object and where, each of which has a corresponding vocabulary, as follows:

- subject: person, hand, product
- verb: move, stay, follow, face to
- object: hand, shelf, cart, product
- where: in the shelf/cart, out of shelf/cart

Subject and object refer to the name of an entity. verb describes the movement of subject or the relation between subect and object. where means the place where the primitive happens. As our proposed method should cover a wide range of CBs, the vocabulary should be a selection of commonly used words in retail environments. Therefore, these words are selected based on our aforementioned findings from the existing methods in Table 1 Nevertheless, more and more words will be available as our research progresses. There are some constraints and options for the syntax to avoid confusing definition sentences, as below:

- subject, verb are required: subject, verb should be filled in. object is required in relation primitives. where is optional.
- Any ignored optional element can be omitted: e.g., if where is ignored, we do not care about the value of where, the syntax can be simplified as subjectverbobject.
- subject≠object: Same subject and object is not allowed in logic.
- The logical operator NOT(!) is allowed: It indicates all words except this one.

In sum, the syntax describes what an object does or what happens to it. With some verbs, it could represent two objects’ relationship. This design could define motion primitives, the motion of an object, relation primitives, or the relation between two objects. In the case of more than two objects, combining several relation primitives could describe a CB composed of multiple objects.

**Table 1:** Primitives in target CBs of current approaches.

Target CB	Related Approaches	Primitives ({} = primitive)
Passing by the Shelf	[3,10,12]	{a person is moving in front of the shelf}
Turning to the Shelf	[10]	{a person is turning to face the shelf}
Viewing the Shelf	[5,10,11]	{a person is standing and watching the shelf}
Touch the Shelf	[3,4,5,10,13]	{one’s hand moves to the shelf},
		{one’s hand moves back from the shelf}
Pick up a Product from the Shelf	[3,4,5,9,10,12,13]	{one’s hand moves to the shelf},
		{one’s hand moves back from the shelf},
		{a product is moving together with one’s hand}
Return a Product back to the Shelf	[3,5,10,12,13]	{one’s hand moves to the shelf},
		{a product is moving together with one’s hand},
		{one’s hand moves back from the shelf}
Put a Product into a Basket/Cart	[10]	{one’s hand moves to the cart},
		{a product is moving together with one’s hand},
		{one’s hand moves back from the cart}
Holding a Product	[11]	{a product is moving together with one’s hand}
Browsing a Product in a Hand	[5,11,13]	{a person is watching his hand},
		{a product is moving together with one’s hand}

However, though the proposed syntax is enough for our current research, its application range is limited due to the design of subject, verb, object, and where. Despite the ability to define multi-object interactions theoretically, each sentence only defines two objects’ one-to-one relationship. Therefore, the resources for multi-object relationships definition grow exponentially with the number of related objects. Nevertheless, it is currently sufficient for us because there are at most two objects in interaction. Since where limits the number of positions only to start and end, it cannot describe complex motion, such as spiral movement.

### 3.2. Primitive Recognition

In this section, we consider the elements in the syntax from the objects’ trajectories. Since most CBs last for a few seconds which implies many frames for a video with 30 fps, this leads to redundancy in the trajectories with the object-tracking method. Consequently, we first perform trajectory segmentation to reduce redundancy in the trajectories. Then, we recognize primitive elements using the results of segmentation.

Trajectory segmentation refers to compressing a trajectory into several segments, which preserve most features of the trajectory. Current approaches [19,20] separate a trajectory based on the moving distance and direction of each vector in the trajectory. Thus, we design an approximate trajectory partitioning (ATP)-based algorithm [19] for trajectory segmentation. However, ATP is sensitive to direction changes. In our case, an object’s frequent direction changes over short distances probably refers to idling. We anticipate that the algorithm will only react to change in the moving distance in this case. Hence, we designed a thresholding algorithm based on ATP as shown in Algorithm 1. The algorithm receives two inputs: a list of points $Kpts_{ATP}\leftarrow \left[p_{1},p_{2},p_{3},...,p_{i},...,p_{N}\right]$ from ATP outputs, where $p_{i}$ refers to the *i*-th element in $Kpts_{ATP}$, *N* is the number of key-points from ATP, and a threshold $threshold_{idle}$ is set to preserve the key-points with a distance longer than $threshold_{idle}$. Since the time complexity of ATP and Algorithm 1 are O(n), the time complexity of the tracjectory segmentation is $O(n^{2})$, where *n* is the length of the trajectory.
**Algorithm 1:** Thresholding Algorithm for Trajectory Segmentation
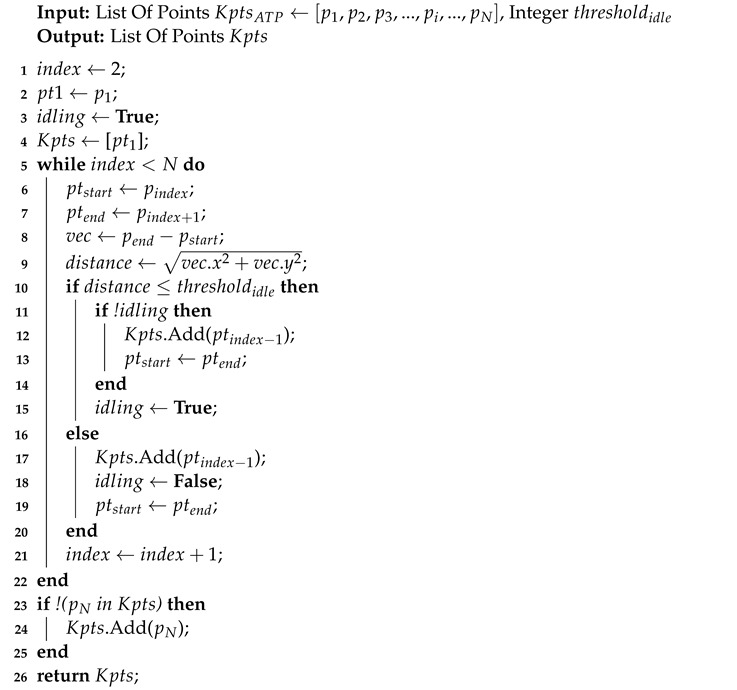


In the primitive’s syntax, subject and object are the entity names that can be obtained directly from the trajectory information. The words “in the shelf/cart” and “out of shelf/cart” for where can be directly acquired from the coordinates of the trajectory. Therefore, only verb needs to be recognized from the trajectories. Algorithm 2 explains the recognition for “move” and “stay”. The two words are a pair of antonyms that mean an object is moving faster than a certain speed or staying still. The input segmented trajectory $ST\leftarrow [p_{1},p_{2},p_{3},...,p_{i},...,p_{M}]$ contains the trajectory processed by segmentation algorithm, where $p_{i}$ refers to the *i*-th point in ST, and *M* is the number of points of ST. $threshold_{idle}$ is reused in this algorithm to detect whether an object is moving or not. To improve the robustness to noise, we applied a window with length of $len_{window1}$ to filter the noise. The algorithm output $verb_{1}$ is one of the words “move” and “stay”, which means the recognition result for the current frame. The time complexity is O(n), where *n* is the smaller of the length of the segmented trajectory and $len_{window1}$.
**Algorithm 2:** Verb Recognition(move, stay)
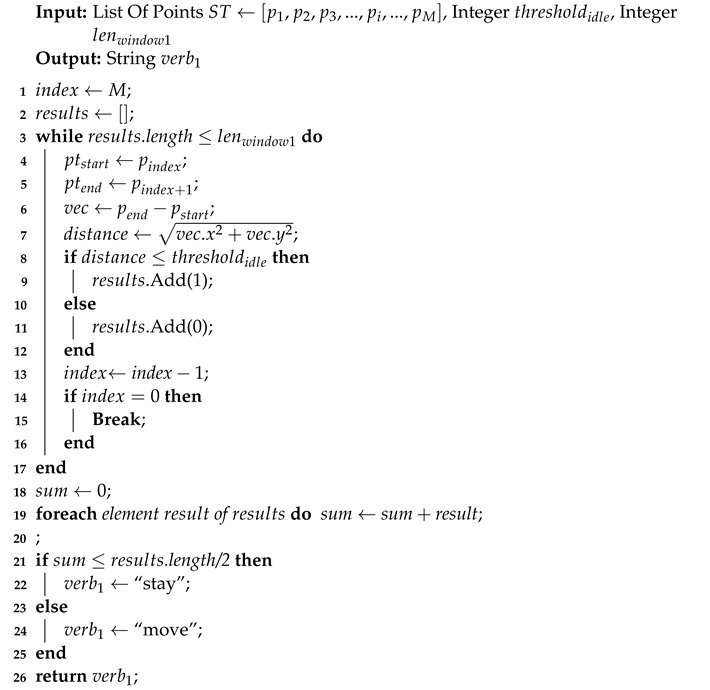


Algorithm 3 shows the recognition for the verb, “follow”. The word means the subject is moving/staying together with the object. The inputs are two objects’ segmented trajectory $ST1\leftarrow [p1_{1},p1_{2},p1_{3},...,p1_{i},...,p1_{M}]$ and $ST2\leftarrow [p2_{1},p2_{2},p2_{3},...,p2_{i},...,p2_{M}]$, where $pj_{i}$ refers to the *i*-th point in the trajectory $ST_{j}$, *M* is the number of points of the segmented trajectory. $threshold_{follow}$ is used to detect whether an object is close to another one or not. Similar to Algorithm 2, a parameter $len_{window2}$ is passed to the algorithm for denoising. The algorithm output $verb_{2}$ is “follow” or null, which means the recognition result for the current frame. The time complexity is O(n). Furthermore, the verb “face to” refers to subject is facing object. Since it requires detecting the orientation of the body or head, which is not currently supported in our method, we intend to omit it in this paper and consider it in future work. The time complexity is O(n), where *n* is the smaller of the length of the segmented trajectory and $len_{window1}$.
**Algorithm 3:** Verb Recognition(follow)
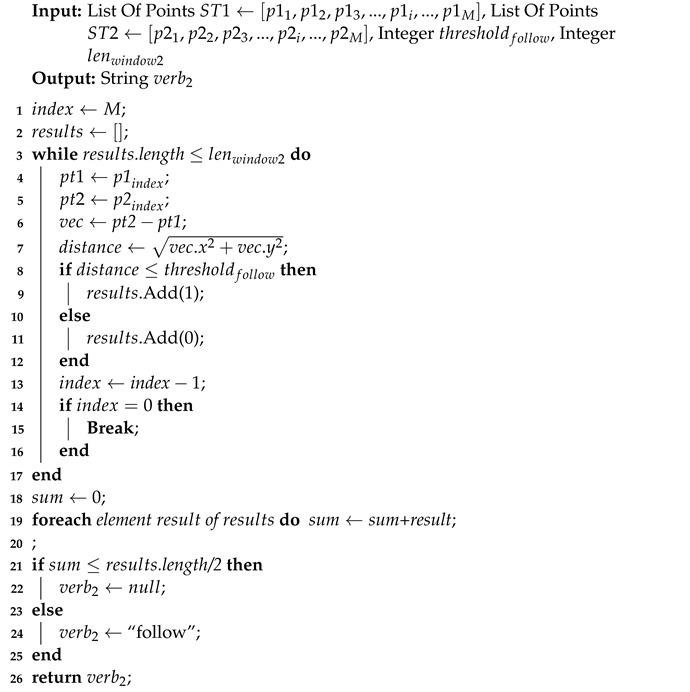


### 3.3. Define CB by Primitives

With our designed primitives, we are able to customize a wide range of CBs with a combination of primitives. Since our primitives are designed with reference to target CBs in existing methods, we applied primitives to define those target CBs. The clothes-related CBs are excepted because they are not common in normal retail stores, and because they are too complex for our proposal. We defined CBs in Table 1 by primitives, as shown in Table 2. The symbol “→” defines the primitives’ chronological order. Primitives that precede this symbol are assumed to occur first. Since the product is occluded when it is on the shelf in our implementation, a precise definition of “touch the shelf” is difficult to formulate. Therefore, we defined it broadly as the primitive pattern in Table 2.

### 3.4. Primitive Pattern Matching

The recognized primitives are stored in a sequence to retain their chronological order. Once any primitive has been recognized in the current frame, our method matches the primitive sequence with the predefined primitive patterns. Any matched result is considered as a recognized CB. Algorithm 4 explains the details of the pattern matching. Since forward matching in chronological order consumes a great deal of computational resources to save different matching states for each primitive pattern, it leads to the running speed becoming slow as the running time grows. Therefore, we match recognized primitives in reverse chronological order. In other words, we start matching from the most recently recognized primitives, which saves a great deal of computational resources because there is no need to save the matching states. The algorithm takes the inputs of a sequence, including recognized primitives, a predefined primitive pattern, and a number timeout, to stop the algorithm when there are not any matched primitives within the recent timeout frames. The output is a Boolean value of whether the corresponding CB is matched or not. The time complexity is O(n), where *n* is the smaller of the length of $P_{seq}$ and the length of $P_{def}$.
**Algorithm 4:** Primitive Pattern Matching
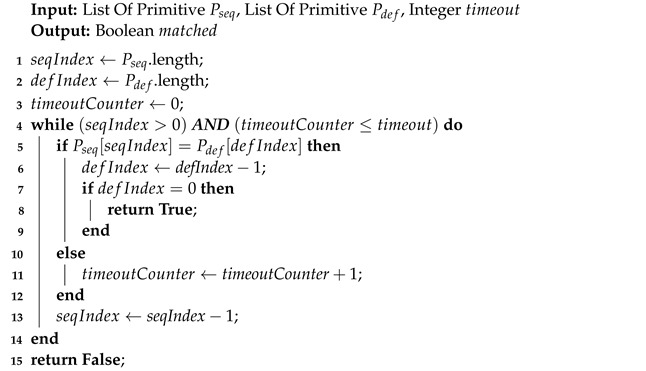


## 4. Evaluation

### 4.1. Experiment Settings

Our proposed method can be flexibly modified to recognize different target CBs to cope with frequently changing target CBs in smart retail solutions. To evaluate our proposed method, we used our collected laboratory dataset [21] and the public MERL dataset [7]. Our proposed method recognizes target CBs in input videos and calculates their f1-score as the accuracy metric. Since videos in two datasets were taken in different environments, it can be considered a change in retail environments to some extent. To recognize different target CBs in the two different datasets, we only changed a few parameters of our designed algorithms and predefined primitive patterns. By observing the accuracy of our method on different datasets, and considering only a few modifications when changing datasets, we could infer our method’s flexibility to some degree.

The inputs of our method are the trajectory coordinates, which need to be obtained using object detection and a tracking model. However, wrong tracking results obtained by other models mean wrong inputs to our method, which probably leads to wrong outputs. To eliminate the influence of different object detection models on our evaluation results, we track the annotated bounding boxes with a Kalman-filter and Hungarian algorithm [22] to obtain the input trajectories for our method. In addition, although some tracking models can predict the trajectories of occluded objects, the occluded trajectories are not annotated in the evaluation. Regarding the output CB annotations, we annotated the target CB in each frame for our laboratory dataset. As the MERL dataset is public, we used its original CB annotations. For the experiments on both datasets, we implemented our method in the same Windows 11 device with RAM of 16 GB. The CPU was an Intel i7-12700K (3.6 GHz). The GPU was an NVIDIA GeForce RTX 3060 Ti (8 GB). The program was written in Python 3.9. The ML framework was PyTorch 1.12. The third-party libraries used included numpy 1.22 and scipy 1.8.

### 4.2. Our Laboratory Dataset

**Figure 2:**
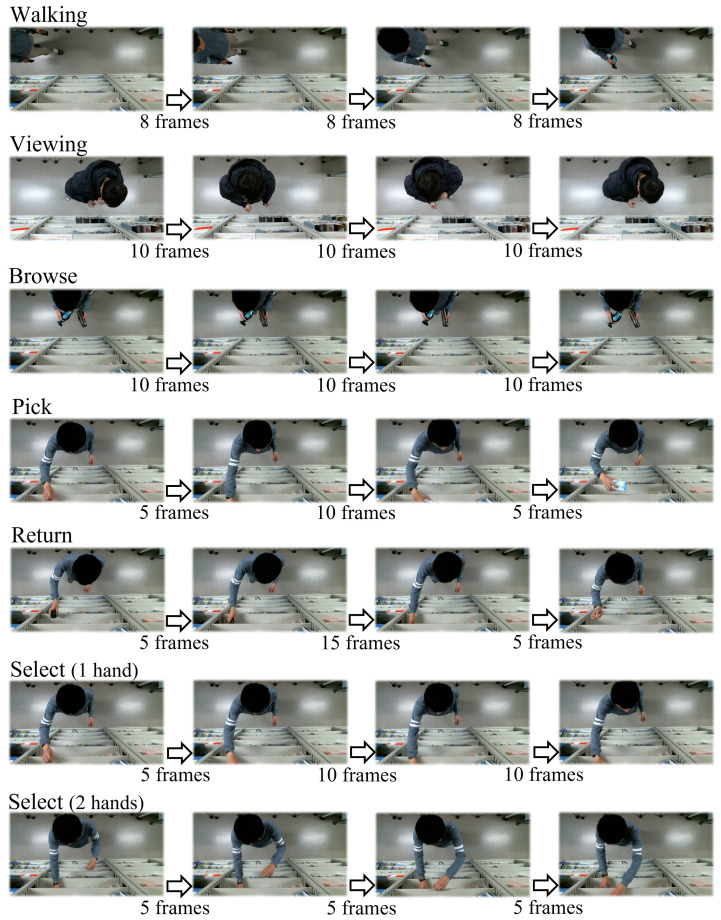
Example of annotated CB in our laboratory dataset.

This is a dataset we collected at a public activity, where the randomly selected 19 participants were requested to simulate shopping in front of the shelf one-by-one. The dataset includes 19 top-view videos of 19 subjects with a resolution of 640 × 480. Each video was about 30–60 s with 10 FPS and only one subject. Figure 2 shows some examples of the annotated target CBs in the dataset. We built a laboratory retail environment and installed an RGB top-view camera to obtain an occlusion-free view. Each participant in the videos was asked to interact with the products on the shelf. The participant were required to take at least one product from the shelf. There were four products of different shapes and sizes, including a boxed juice, a deodorant spray, a stainless steel bottle, and a wet-tissue. The products were not visible when they were on the shelf. Our data was collected when our proposed method was demonstrated in a public activity. The videos were collected without requiring the participants to sign any confidentiality agreement, and the participants’ faces were exposed to the cameras. Unfortunately, as a result, we cannot publish our collected dataset until all the private information has been removed, such as by masking the faces.

Since the innovative part of our proposed method involves the receipt of trajectory coordinates as inputs, we annotated the bounding box of person, hand, and four products in each frame. Then, we used a tracker with a Kalman-filter and Hungarian algorithm [22] to obtain the object’s trajectory as input. Regarding the output CBs, we selected eight CBs as listed in Table 3. Among them, the first six CBs included most target CBs used in existing methods. However, with the annotation of the first six CBs, we found that many frames still remained without annotation. Thus, we added two CBs to fill the frames without annotations. We used some approximate definitions for some CBs, such as “browse”, because the approximate definition enabled reuse of primitives with nearly no loss of accuracy.

**Figure 3:**
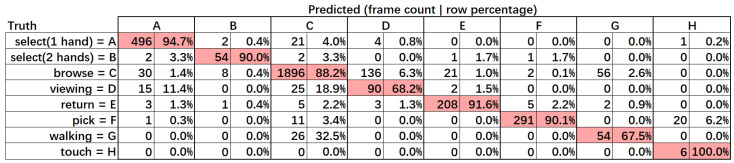
Confusion matrix of our laboratory dataset.

**Figure 4:**
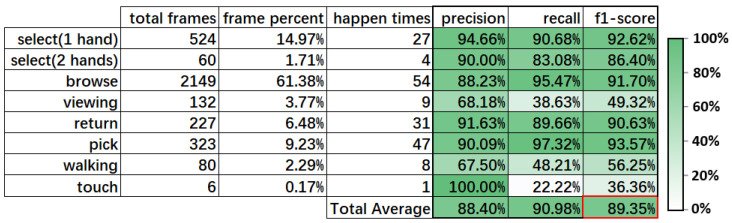
Results of F1-score of our laboratory dataset.

Figure 3 shows the confusion matrix of our laboratory dataset. Each CB’s column includes two columns of frame count and each row’s frame count percentage. Figure 4 shows the f1-score and some statistics for our laboratory dataset. The total average is the average value of the column calculated using the sum of the product of the frame percent and each row’s value. The total average f1-score of our method was 89.35%, which is an acceptable result. The f1-score for most CBs was also acceptable, except for “viewing,” “walking,” and “touch”. In terms of “viewing”, the confusion matrix revealed the reason with 68.18% precision. Some “viewing” frames were recognized as “select” and “browse.” The ambiguous boundary caused the wrong prediction of “select”. The different definition of “viewing” between annotation and CB definition led to the wrong prediction of “browse”. As our proposed method cannot recognize the target’s orientation or track the target’s eyes currently, our CB definition approximately defines “viewing” as stay static out of the shelf, while the annotation of “viewing” means the target is standing still and looking at the shelf. The low recall of “viewing” indicates that most frames of “browse” were recognized as “viewing”. The difference in CB definition is whether the target is holding a product or not. Products are usually occluded in the “browse” frames, which caused the wrong recognition output for “viewing”.

With respect to “walking”, some of its frames were recognized as “browse”. When the target is walking while holding a product, it is difficult to determine the ambiguous boundary between “browse” and “walking”. The CB definition in Table 3 recognizes them by distinguishing whether the target is moving while holding a product. “Browse” refers to holding a product while staying static. We used a single threshold to divide the object’s moving speed to detect move or stay, which was not sufficiently accurate for totally correct detection. Some frames were detected as staying static, which led to the wrong recognition. This also applied to the low recall of “walking”.

In the case of “touch”, there was only one case in the dataset. It was defined as a customer putting their hand inside the shelf but taking nothing out of it. Some wrong recognition of “pick” results in the low recall occurred because the picked object was occluded. In addition, Figure 3 shows that most video frames were “browse” and occurred more frequently than any other CBs. Thus, we considered discriminating within “browse” to make the distribution of CBs more uniform.

According to the above results, our method showed acceptable accuracy for the laboratory dataset. Some individual CBs with low f1-score are anticipated to be improved by changing the CB definitions into more accurate definitions. To evaluate our proposed method’s ability to discriminate CB, we predefined different primitive patterns to discriminate the CB “select” according to whether one hand or both hands were used. This indicates that our proposed method is able to deal with CB discrimination to some extent. Concerning the evaluation of flexibility, we measured the time required by our method when applied to different datasets. For the collected laboratory dataset, we spent about an hour tuning the five parameters in the three designed algorithms and two to three hours defining the primitive patterns in Table 3. Then, we annotated the CBs in each frame for about five hours per day. The annotations took about one week in total. Since annotation is not required during the application of our method, the time for annotation is considered as a reference for the ML-based methods’ modification time.

### 4.3. MERL Dataset

**Figure 5:**
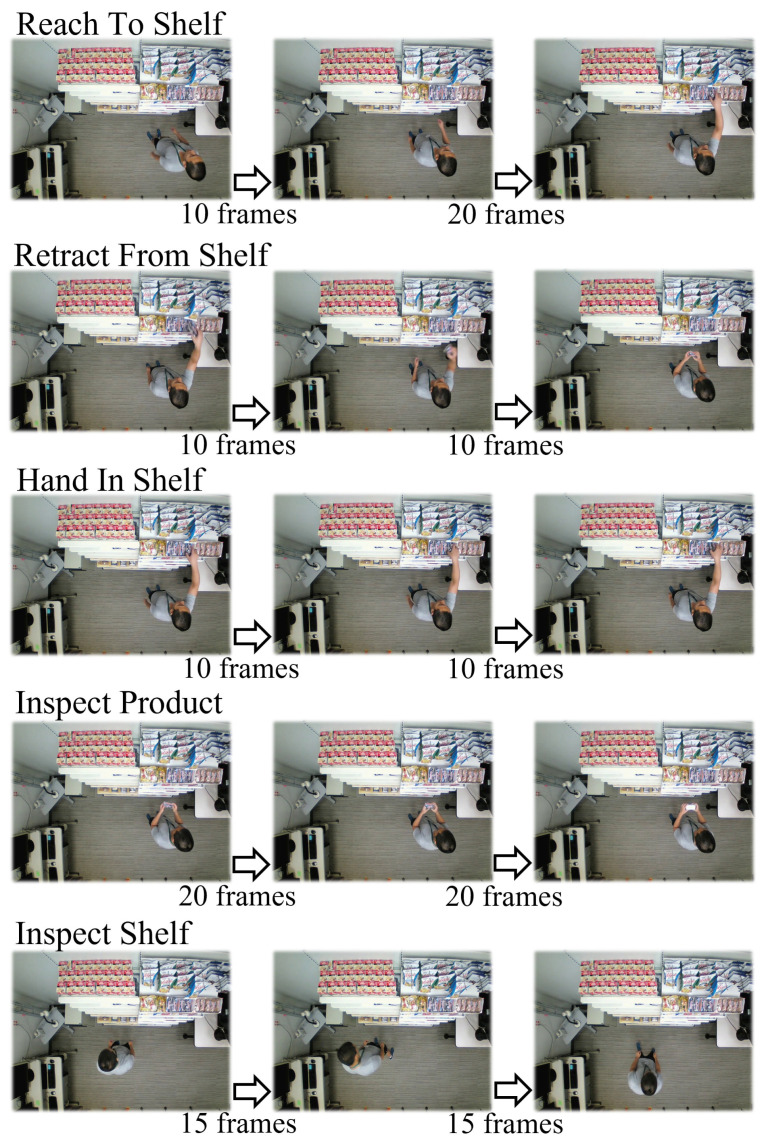
Example of annotated CB in MERL dataset.

The MERL shopping dataset [7] is a public dataset consisting of 106 top-view videos with a resolution of 920 × 680, each of which is about two minutes long with 30FPS. All 41 subjects were asked to do shopping in a retail store setting. Figure 5 presents some examples of the annotated CBs in the dataset. With regard to the input trajectory coordinates, we annotated the bounding box of person and hand in each frame based on the results from the pose estimation model Higher HRNet [23] pretrained on the COCO dataset [24]. We manually annotated the product’s bounding box in each frame. Due to the limited time, we only finished the object’s bounding box annotations in 46 videos for evaluation. Similar to the process for the laboratory dataset, we used the same tracker with a Kalman-filter and Hungarian algorithm [22] to obtain the input trajectories.

For the output CBs, we used the CB annotations included in the dataset. This provided five CBs’ annotation, and we defined them using our proposed method, presented in Table 4. Among the five CBs, we excluded the CB “hand in shelf” from the evaluation because many ground truths were not annotated during our random check of the annotations.

**Figure 6:**
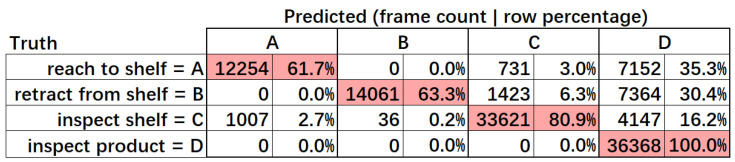
Confusion matrix of MERL dataset.

**Figure 7:**
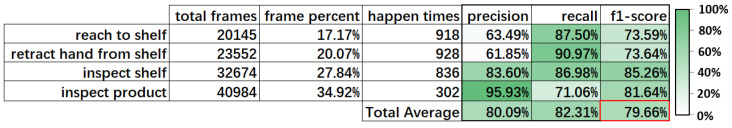
Results of F1-score of MERL dataset.

Figure 6 shows the confusion matrix of the MERL dataset. Each CB’s column includes two columns of frame count and each row’s frame count percentage. Figure 7 shows the f1-score and statistics for the MERL dataset. The calculation of the total average was the same as in Figure 4. The average f1-score of our method was 79.66%, which is acceptable for our proposed method with only a change in CB definitions. Among the four target CBs, our method achieved only about 60% precision for “reach to shelf” and “retract from shelf”. We found that this was caused by the different boundary in the definition. Specifically, there was a difference between our definition of “reach to shelf” and the definition in the MERL dataset. We defined the CB’s boundary using a threshold of moving speed. Therefore, our method started to recognize “reach to shelf” from the frame in which the hand was already moving. The MERL dataset defines the start of “reach to shelf” as when one intends to “reach to shelf”, when one’s hand has not yet moved. Thus, our recognition results always differed from the annotations by a few frames. For “retract from shelf”, this accounted for the low precision. The errors for “reach to shelf” and “retract from shelf” were caused by different definitions. We consider our method to have been successful in recognizing every “reach to shelf” and “retract from shelf” CB with a few frames’ difference. This implies that we could improve our method by recognizing intention in our future research.

Except for recognition accuracy, Table 5 compares the required modifications and the estimated required time when applying our approach and the machine learning-based approach to different datasets. Our proposed method changed the five parameters ($threshold_{idle}$, $len_{window1}$, $threshold_{follow}$, $len_{window2}$, timeout) in the three algorithms we designed in Section 3. They were mainly used to cope with change in the person’s scale in the video frames. We also re-defined the primitive patterns for the new target CBs. As shown in Table 5, in our experiments, all the modifications took about 3–4 h.

For the ML-based methods, the main modification was re-annotation. Since the required time for data re-collection and model tuning varied greatly when dealing with changes of datasets, we currently lack sufficient reference data to estimate its required time. However, regarding the time spent on re-annotation, as we annotated both datasets for the purpose of accuracy calculation, the required time for modification was estimated to be about 2–3 months.

In conclusion, since our method cannot be fine-tuned as ML-based methods are, our proposed method sacrifices accuracy to obtain flexibility. Nonetheless, the huge difference in modification time indicates that the trade-off is justified. The considerably enhanced flexibility could have application value in the context of CBR.

## 5. Conclusions

Smart retail solutions usually require the recognition of a wide range of CBs from captured video in stores. The CBs that are selected as recognition targets are called target CBs. Target CBs frequently change with changes in needs, environments, etc. To achieve flexible target CB change adaptation, we proposed a flexible CBR approach. Our main idea is recognizing CB using a combination of primitives, which are a kind of partitioned CB. Since different CBs share the same primitives; the primitives can be reused when adapting to target CB changes, which avoids time-consuming steps, such as re-collecting training data and re-training the recognition models. Consequently, our method can flexibly adapt to changes in target CB by changing the combinations of primitives only. In addition, we designed a syntax based on natural language grammar to define primitives. The readable syntax improves the explanatory power of our method. Therefore, the usage of primitives and our proposed syntax can enable a high degree of flexibility in target CB change adaptation. Evaluation experiments undertaken demonstrated that our method achieved an acceptable level of accuracy for different datasets, and great flexibility across different datasets.

Nevertheless, the experiments also revealed some limitations of our proposed method. Since our method is difficult to fine-tune to fit some individual situations, the recognition accuracy is decreased compared to ML-based methods. A possible solution would be to replace the current pattern matching algorithm with a probabilistic model. In addition, because the element where in the primitive syntax limits the number of positions, the syntax cannot represent complex movement, such as spiral movement. This leads to a limited cover range of CB. Increasing the vocabulary of where could improve the model’s expressive power to represent complex movement. Furthermore, though the syntax element faceto includes orientation information, the orientation detection is currently not applied. These limitations may be addressed in future work.

## Figures and Tables

**Table 2:** Define target CBs by primitives.

Target CB	Primitive Pattern ({} = Primitive, != Not)
Passing by the Shelf	{person move out of shelf}
Turning to the Shelf	{person !face to shelf} → {person face to shelf}
Viewing the Shelf	{person stay out of shelf}
Touch product in the Shelf	{hand move to in the shelf} →
	{hand move to out of shelf}
Pick up a Product from the Shelf	{hand move to in the shelf} →
	{hand move to out of shelf}, {product follow hand}
Return a Product back to Shelf	{hand move to in the shelf}, {product follow hand}
	→ {hand move to out of shelf}
Put a Product into a Basket/Cart	{hand move to in the cart}, {product follow hand}
	→ {hand move to !in the cart}
Holding a Product	{product follow hand}
Browsing a Product in a Hand	{product follow hand}, {person stay}

**Table 3:** Primitive patterns in our laboratory dataset.

Name	Description	Primitive Pattern ({}= Primitive, != Not)
Walking	one is walking along the shelf	{person move out of shelf}
Viewing	one is standing in front of the shelf	{person stay out of shelf}
Browse	one is holding and watching a product without moving	{product follow hand}, {person stay}
Pick	one picks up a product from the shelf	{hand move to in the shelf} →
		{hand move to out of shelf}, {product follow hand}
Return	one returns a product back to the shelf	{hand move to in the shelf}, {product follow hand}
		→ {hand move to out of shelf}
Touch	one hand into the shelf without taking any product out	{hand move to in the shelf} →
		{hand move to out of shelf}
Select (1 hand)	one hand is selecting products in the shelf	{hand in the shelf}, {hand out of shelf}
Select (2 hands)	both hands are used to select products in the shelf	{hand out of shelf}

**Table 4:** Primitive Patterns in MERL dataset.

Name	Description	Primitive Pattern ({}= Primitive)
Reach To Shelf	reach one’s hand to shelf	{hand move out of shelf} →
		{hand move in the shelf}
Retract From Shelf	retract hand from shelf	{hand move in the shelf} →
		{hand move out of shelf}
Hand In Shelf	extended period with hand in the shelf	{hand in the shelf}
Inspect Product	inspect product while holding it in hand	{product follow hand}
Inspect Shelf	look at shelf while not touching and reaching for the shelf	{person stay out of shelf}

**Table 5:** Flexibility: Modifications for dataset change adaptation.

Method	Modifications	Estimated Required Time
Our proposed method	5 parameters for our designed 3 algorithms	1 h
	Re-define primitive patterns	2–3 h
	Re-collecting video data	no reference data
ML-based methods	Re-annotating collected data	a week (our dataset)
		3 months (MERL)
	Training and tuning model(s)	no reference data

## Data Availability

Not applicable.

## Abbreviations

The following abbreviations are used in this manuscript:

CB	Customer behavior
CBR	Customer behavior recognition
HAR	Human activity recognition
ML	Machine learning
